# Experimental on-farm trials data of faba bean and wheat intercropping field validation in Lebanon and Morocco

**DOI:** 10.1016/j.dib.2022.108098

**Published:** 2022-03-24

**Authors:** Fouad Maalouf, Lynn Abou Khater, Shiv Kumar, Kamal Hejjaoui, Walaa Morda, Perla Hayek, Lamis Chalak, Asma Jeitani, Pietro Bartolini

**Affiliations:** a International Center for Agricultural Research in the Dry Areas, ICARDA; b Lebanese University, Faculty of Agricultural Engineering and Veterinary Medicine Lebanese

**Keywords:** Faba bean, Wheat, Crop mixture, Dry areas, Breeding for intercropping

## Abstract

This data paper describes the content of four datasets collected by the International Center for Agricultural Research in the Dry Areas (ICARDA) as a partner in the project “Designing InnoVative plant teams for Ecosystem Resilience and agricultural Sustainability (DIVERSify)” with the objective of assessing the feasibility of faba bean-wheat mixture in Mediterranean environments under diverse rainfed conditions. Data was collected during the trials conducted in Kfardan-Lebanon during 2017/2018 where 40 faba bean varieties were evaluated as sole and as mixture with 2 wheat cultivars ‘Margherita’ and ‘Miki’ and during 2018/2019 where 40 faba bean varieties and one durum wheat cultivar ‘Margherita’ were evaluated under low rainfall environments. Trials were also conducted in Tal Amara-Lebanon during 2019/2020 where 20 faba bean lines and one durum wheat cultivar ‘Margherita’ were evaluated under high rainfall environments and in Marchouch-Morocco during 2019/2020 where 7 faba bean lines with 3 cultivars and one durum wheat cultivar ‘Margherita’ were evaluated under extremely low rainfall environments. A detailed list of the different biological traits collected for wheat and faba bean is found in the specification table in this article. The Kfardan 2018/ 2019, Tal Amara and Marchouch data is related to the conference paper “Performance of faba bean-wheat mixture under diverse Mediterranean environments” [1].

## Specifications Table

**Table:** 

Subject	Agronomy and Crop Science
Specific subject area	Selection of faba bean accessions intercropped with wheat plant in the diverse dry regions of Lebanon and Morocco
Type of data	Tables, images
How data were acquired	Field data were collected directly from experimental field conducted in different seasons and locations. The data was entered to eBook using electronic tablet such as days of flowering and maturity, plant height, The laboratory data were taken using precise weighting scale for measuring the yield and seed counter to estimate hundred seed weight in faba bean and thousand kernel weights. The field maps were prepared in power point and uploaded as images.
Data format	Raw
Description of data collection	The faba bean data were recorded either in the field or in the laboratory at Terbol station using faba bean ontology [2]. In the field, the following data were recorded: Days to Flowering (DFLR) at 50% flowering time, Days to Maturity (DMAT) at 50% flowering time, Plant Height (PTHT), First Pod Height (FPHT), Number of Branches per Plant (NBPP), Number of Pods per Plant (NPP), Number of Pods per First Node (NPFN) and Number of weeds in each plot before weeding. In the laboratory, the following data were recorded Hundred Seed Weight (HSW g), Grain Yield (GY kg/ha), Biological Yield (BY kg/ha), Grain Yield (GY), The wheat data recorded in the field were Days to Heading (HD), Days to Maturity (DMAT), Plant Height (PTHT) and Tillage in three independent plants while other data were recorded in the laboratory are grain yield and thousand Kernel Weight, Combined yield and biological yield per ha for both crops were recorded
Data source location	Institution: Lebanese Agricultural Research Institute station City/Town/Region: Kfardan Country: Lebanon Latitude and longitude for collected data: Long 36.04647222 and Lat 34.00713888 Institution: Lebanese Agricultural Research Institute station City/Town/Region: Tal Amara Country: Lebanon Latitude and longitude for collected data: Long 36.5 and Lat33.46666 Institution: International Center for Agricultural Research in the Dry Areas research station City/Town/Region: Marchouch Country: Morocco Latitude and longitude for collected data: Long 33.5581 and Lat -6.693
Data accessibility	All 4 datasets are available as open access files on our MEL repository: **MEL dataverse,** The identifier number of 4 data sets were: 20.500.11766.1/FK2/MHOHHL, 20.500.11766.1/FK2/O5YFWX, 20.500.11766.1/FK2/MQRV9A 20.500.11766.1/FK2/HTBOKU. The links of four data sets are respectively
	DIVERSify field experiment results in Kfardan 2018: link: https://hdl.handle.net/20.500.11766.1/FK2/MHOHHL, DIVERSify field experiment results in Kfardan 2019: https://hdl.handle.net/20.500.11766.1/FK2/O5YFWX DIVERSify field experiment results in Marchouch 2020: https://hdl.handle.net/20.500.11766.1/FK2/MQRV9A DIVERSify field experiment results in Tal Amara 2020: https://hdl.handle.net/20.500.11766.1/FK2/HTBOKU

## Value of the Data

• These data provide information regarding the performance of intercropping vs monocropping in Dry environments. This data helps to identify suitable combinations Faba bean + wheat that yield better under tested environments.
• This data will benefit researchers working on wheat - faba bean systems, crop modelers, breeders interested in selecting accessions for intercropping and farmers.
• The data can be used as a base for crop modeling and breeding for intercropping for any other project dealing with plant team in North and West Asia.

## 1 Data Description

This raw data in brief describes four datasets collected by ICARDA as a partner in the project “Designing InnoVative plant teams for Ecosystem Resilience and agricultural Sustainability (DIVERSify)”.

The datasets “DIVERSify field experiment results in Kfardan 2018” and “DIVERSify field experiment results in Kfardan 2019” contain experimental data collected in Kfardan (Lebanon) in 2018 and 2019 respectively. Datasets “DIVERSify field experiment results in Tal Amara 2020” and “DIVERSify field experiment results in Marchouch 2020” contain experimental data collected in 2020 in Tal Amara (Lebanon) and Marchouch (Morocco) respectively.

Each dataset comprises 8 or 9 CSV files and a pdf file that displays an image of the field plan of the relevant trial. The content of each CSV file is summarized in Table 1.

**Table 1:** Datasets Content Summary.

CSV File Name	File General Description	Variables
Data Dictionary: Introduction	The file provides background explanatory information about the dataset.	Description, Summary, Start Date, End Date, Authors, Co-authors.
Data Dictionary: Element Description	The file provides explanation for each variable/column and any code used inside the dataset.	Element Display Name, Description, Unit, Data Type etc.
Data Dictionary: Unique Identifier	The file provides reference links to an online resource for elements, terms, and concepts used in the dataset.	Element Display Name, Unique Identifier, Source etc.
01 Plot information	The file contains data on plot locations and information about "what was grown where".	Plot Code, Site, Crop Species Common Name, Variety Combination, Number Of Plant Species etc.
02 Plot level data	The file contains all measurements (raw data) taken at the level of a whole plot.	Canopy height etc.
03 Species level data	The file contains all measurements (raw data) taken for each crop species per plot (for example the legume partner), but without information on which plant individual was measured.	Grain Yield etc.
04 Individual level data	The file contains all measurements (raw data) taken at the level of individuals Note: File found in datasets Kfardan 2019 and Tal Amara 2020 only.	Number of branches etc.
05 Metadata Field	The file contains data on site management and description.	Earliest sowing date, Major Herbicides (mixture), Major Insecticides (Legumes) etc.
06 Field plan	The file contains the spatial distribution of the plots planted in the field	Spatial position of row and column of each planted plots is determined. It contains plot numbers

## 2 Experimental Design, Materials and Methods

### 2.1 Plant material and experimental design

#### 2.1.1 Kfardan 2018

The field experiment was located in the Lebanese Agricultural Research Institute station in Kfardan Lebanon in 2018. The trial included 40 faba bean varieties and 2 wheat cultivars ‘Margherita’ and ‘Miki’. It was conducted in a spatial row/column design with 2 replications and 5 treatments: the first is Faba bean monoculture, the second is Wheat sole (first variety), the third is wheat sole (second variety), the fourth is mixture of Faba bean/Durum Wheat (first variety) and the fifth is mixture of Faba bean/Durum Wheat (second variety). The length of individual plot is 5 m and the width of individual plot is 1.2 m (4 rows x 5m).

#### 2.1.2 Kfardan 2019

The field experiment was located in the Lebanese Agricultural Research Institute station in Kfardan Lebanon in 2019. The trial included 40 faba bean varieties and one durum wheat cultivar ‘Margherita’ evaluated under low rainfall environments. It was conducted in a spatial row/column design with 3 replications and two treatments: the first is Faba bean monoculture, the second is mixture of Faba bean/Durum Wheat. The length of individual plot is 5m and the width of individual plot is 1.2 m (4 rows x 5m).

#### 2.1.3 Tal amara 2020

The field experiment was located in the Lebanese Agricultural Research Institute station in Tal Amara Lebanon in 2020. The trial included 20 faba bean lines and one durum wheat cultivar ‘Margherita’ evaluated under high rainfall environments. It was conducted in a spatial row/column design with 3 replications and four treatments: the first is Faba bean monoculture, the second is mixture of 100% Faba bean / 100% Durum Wheat, the third is mixture 100% Faba bean 50% Durum wheat, the fourth is wheat monoculture. The length of individual plot is 5m and the width of individual plot is 1.2 m (4 rows x 5m).

#### 2.1.4 Marchouch 2020

The field experiment was located in ICARDA's research station in Marchouch, Morocco in 2020. The trial included seven faba bean lines with three cultivars and one durum wheat cultivar ‘Margherita’ evaluated under extremely low rainfall environments. It was conducted in a spatial row/column design with 2 replications and four treatments: the first is Faba bean monoculture, the second is mixture of 100% Faba bean / 100% Durum Wheat, the third is mixture 100% Faba bean 50% Durum wheat, the fourth is wheat monoculture. The length of individual plot is 5 m and the Width of individual plot is 3 m (4 rows x 5m).

### 2.2 Data collection

Traits recorded in faba bean were as follows: Days to flowering (DFLR), Days to maturity (DMAT), Plant height (FBPLHT in cm), First pod height (FPHT in cm), Number of branches per plant (NBPP), Number of pods per plant (NPPLT), Number of seeds per plant (NSPLT), Number of seeds per pod (NSPP), Hundred seed weight (HSW in g) and Grain yield (FBGY in kg/ha).

The recorded traits in wheat were as follows: Days to heading (HD), Plant height (WPLHT in cm), Tillage (TPL): Average number of shoots of three plants per plot, Grain yield (WGY): weight of kernels harvested in kg/ha, Thousand Kernel weight (TKW). The combined traits were total biological yield (BY in kg/ha) and GY in kg/ha.

Phenological data, number of pods per plant, plant height and number of branches were recorded in the field while yield and yield components data (seed number, hundred seed weight) were recorded in the laboratory at Terbol station. Data was collected by the technical staff in Terbol and Marchouch in addition to the students working on their master's thesis, using electronic books. The datasets were curated following ICARDA's General Dataset Curation Guide (GDCG) [3].

## Ethics Statement

The authors declare that they have no known competing financial interests or personal relationships which have or could be perceived to have influenced the work reported in this article.

## CRediT Author Statement

**F. Maalouf:** Conceptualization, Methodology, Data collection, Writing – review & editing, Supervision; **L. Abou Khater** and **K. Hejjaoui:** Data collection, Writing; **L. Chalak, W. Morda** and **P. Hayek:** Two Master thesis developed, methodology and data collection; **P. Bartolini** and **A. Jeitani:** Dataset curation, Writing.

## Declaration of Competing Interest

The authors declare that they have no known competing financial interests or personal relationships which have or could be perceived to have influenced the work reported in this article.

## Data Availability

DIVERSify field experiment results in Tal Amara 2020 (Original data) (Dataverse). DIVERSify field experiment results in Marchouch 2020 (Original data) (Dataverse). DIVERSify field experiment results in Kfardan 2019 (Original data) (Dataverse). DIVERSify field experiment results in Kfardan 2018 (Original data) (Dataverse).
